# Correlation between clinical and pathological data and surgical margins in patients with squamous cell carcinoma of the oral cavity

**DOI:** 10.5935/1808-8694.20130034

**Published:** 2015-11-02

**Authors:** Fábio Muradás Girardi, Virgílio Gonzales Zanella, Ricardo Galicchio Kroef

**Affiliations:** a Head and Neck Surgeon at the Santa Rita Hospital, Santa Casa Hospital Complex of Porto Alegre, Brazil; b Head of the Head and Neck Surgery Service and Medical Director of the Santa Rita Hospital. Head and Neck Surgery Department, Hospital Santa Rita, Complexo Hospitalar Santa Casa de Porto Alegre

**Keywords:** mouth neoplasms, neoplasms, surgery, oral, tongue neoplasms

## Abstract

The importance of having tumor-free margins when resecting oral neoplasms has been known for decades.

**Objective:**

To correlate clinical and pathology data to surgical margin status in patients with squamous cell carcinoma of the tongue and floor of the mouth.

**Method:**

This historical cohort cross-sectional study included all patients submitted to squamous cell carcinoma resection for tumors of the oral tongue and floor of the mouth between 2007 and 2011 at the Head and Neck Surgery service of our institution.

**Results:**

In the 117 cases included, 68.3% had tongue tumors. The male-to-female ratio was 2.3:1 and patient mean age was 57.6 years. Broad free resection margins were seen in 23.0% of the cases; narrow margins in 60.6% of the cases; and compromised margins in 16.2%. Tumor diameter and thickness were correlated to resection margins. Tumors in more advanced T-stages presented more unsatisfactory margins. Patients operated with broad free margins had their tumors resected more commonly through transoral approaches.

**Conclusions:**

Tumors of larger volume both in terms of diameter and thickness were more correlated to unsatisfactory resection margins. Higher complexity procedures were not associated with better resection margins.

## INTRODUCTION

Oral cancer ranks fifth among malignant neoplasms affecting men in Brazil^1^. In the past few decades, not only incidence rates but also the number of deaths caused by this disease have increased in Brazil^2^. Oral cancer is primarily treated with surgery. The importance of obtaining tumor-free margins when treating squamous cell carcinoma (SCC) of the mouth has been known for decades^3^. Unsatisfactory margins have been correlated with local relapsing tumors and decreased patient survival^3^.

Associations between involved margins and factors related to oral cancer patient survival such as T-stage^3^, ^4^, ^5^, ^6^, ^7^, ^8^, N-stage^5^, ^6^, thickness^9^, and pattern of tumor invasion^5^, ^10^, ^11^ have been reported. However, most authors tend to group together patients with tumors of all areas of the mouth^12^, ^13^, ^14^, ^15^ without analyzing specific sites or sub-groups, in addition to adopting a wide array of criteria to define margin status^13^, ^15^, ^16^. The ability of the surgeon to obtain disease-free margins may be affected by the location of the tumor^6^. Loree & Strong^3^ reported significant variations in involved margin incidence for different sites in the mouth.

Tumors of the oral tongue and floor of mouth account for 41% to 74% of the squamous cell carcinomas of the mouth^7^, ^8^. The way they progress and are treated surgically are quite similar. This paper aimed to correlate histopathology findings to surgical margin status of patients with SCC of the oral tongue and floor of the mouth.

## METHOD

The study was approved by the Research Ethics Committee of the institution. This historical cohort cross-sectional study included all patients submitted to resection of malignant tumors of the oral cavity between 2007 and 2011 at the Head and Neck Surgery service of the hospital. A total of 385 cases were found, 333 (86.4%) of which with SCC. One hundred and fifty-three (45.9%) had SCC of the oral tongue and floor of the mouth. Seventeen cases were excluded for lacking histopathology data on tumor diameter and thickness, 17 for having undergone head and neck radiation therapy or surgery involving the oral cavity, and two for having had palliative surgery. Ultimately, the study enrolled 117 cases, featuring all untreated patients with SCC of the oral tongue and floor of the mouth submitted to curative surgery in that period.

Histopathology data, age, gender, and surgical approaches were analyzed. The data was collected by one researcher (Girardi FM). Statistical analysis was carried out using software EpiInfo version 3.4.3, 2007. Descriptive analysis was used to summarize the data. Variables were expressed in terms of frequencies, mean values and standard deviations as needed. Tumor diameter and thickness presented a normal distribution, as verified by the Anderson-Darling test. The *t-test* was used to compare the mean values of continuous variables and the chi-square test to compare the frequencies of qualitative variables. An alpha value of 0.05 was considered in all tests.

Tumor staging was performed in accordance with the sixth edition of the staging system of the American Joint Committee on Cancer pTNM^17^. The distance between tumor cells and surgical margins was measured using an ocular micrometer. Surgical margins were defined as described in Batsakis^18^: free margins, when the tumor is at least 5 mm away from the surgical margin; narrow margins, when the tumor is less than 5 mm away from the surgical margin; and involved margins, when tumor is present in the surgical margin. Broadening of surgical margins performed during surgery or after the primary procedure were not considered in the calculation and categorization of the surgical margins. Involved and narrow tumor resection margins were categorized as unsatisfactory margins. Surgical approaches were categorized as transoral (TO) or complex surgery, including labiotomy with or without resection or sectioning of the mandible, and visor flap transcervical approaches. Only two groups were compared in statistical analysis: patients with satisfactory and unsatisfactory surgical resection margins.

## RESULTS

Eighty (68.3%) of the 117 selected cases had SCC of the tongue, and 37 (31.6%) had SCC of the floor of the mouth. The male-to-female ratio was 2.3:1. Subject mean age was 57.6 ± 14.5 years, ranging from 13 to 95. No correlation was seen between mean age and surgical margin status (*p* = 0.8737). Mean top tumor diameter was 2.4 ± 1.3 cm, ranging from 0.3 to 7.8 cm. Mean top tumor thickness was 1.0 ± 0.7 cm, ranging from 0.1 to 3.5 cm. Ninety (76,9%) patients underwent neck dissection, and 38 (32.4%) had metastatic nodes. Twenty-seven subjects (23.0%) had broad free resection margins, 71 (60.6%) had narrow margins, and 19 (16.2%) had margins involved by tumor. Distributions by grade of tumor differentiation, neurovascular and node invasion, T and N stages, surgical margins, surgical approach, tumor diameter and thickness are shown in Table 1.

**Table 1:** Descriptive analysis of clinical and histopathology characteristics related to margin status (involved, narrow, or free).

Margin status*	A		B		C		Total	
	n	%	n	%	n	%	n	%
Gender								
Male	12	63.1	53	74.6	17	62.9	82	70.0
Female	7	36.8	18	25.3	10	37.0	35	30.0
Differentiation grade								
WD	0	0	20	28.1	8	29.6	28	23.9
MD	14	73.6	35	49.2	14	51.8	63	53.8
NVD	4	21.0	10	14.0	2	7.4	16	13.6
Basaloid	1	5.2	0	0	0	0	1	0.8
Verrucous	0	0	2	2.8	0	0	2	1.7
Microinvasive	0	0	4	5.6	3	11.1	7	5.9
NVN invasion	5	26.3	13	18.3	4	14.8	22	18.8
T-stage								
T1	3	15.7	33	46.4	16	59.2	52	44.4
T2	7	36.8	18	25.3	10	37.0	25	21.3
T3	3	15.7	7	9.8	0	0	10	8.5
T4	6	31.5	13	18.3	1	3.7	20	17.0
N-stage**								
N+	8	42.1	22	30.9	7	25.9	37	31.6
N-	11	57.8	49	69.0	20	74.0	80	68.3
Surgical approach								
TO	11	57.8	48	67.6	24	88.8	83	70.9
Complex	7	36.8	18	25.3	1	3.7	26	22.2
NI	1	5.2	5	7.0	2	7.4	8	6.8
μ tumor diameter***	3.38	-	2.33	-	1.97	-	2.42	-
μ tumor thickness***	1,51	-	1,07	-	0,69	-	1,05	-
Total	19	100	71	100	27	100	117	100

^*^ A: Involved margin; B: Narrow margin; C: Free margin;

^**^ N+/N-: Histopathology with positive or negative nodes;

^***^ Mean values in cm; NVN: Neurovascular or nodal; TO: Transoral; WD: Well-differentiated; MD: Moderately differentiated; NVD: Not very differentiated; NI: Not informed.

Tumor diameter and thickness were correlated to surgical resection margins. Larger tumors and more advanced T-stages had more unsatisfactory margins (Tables 1, 2, and 3). However, 72.4% (42/58) of the tumors of two centimeters or less also had unsatisfactory margins (Table 2).

**Table 2:** Descriptive analysis and correlation between surgical margin status and tumor diameter.

Margin status*		A		B		C
	N	%	N	%	N	%
Diameter						
≤ 1 cm	0	0	11	15.4	6	22.2
1.1-2 cm	4	21.0	27	38.0	10	37.0
2.1-3 cm	7	36.8	14	19.7	7	25.9
3.1-4 cm	2	10.5	10	14.0	4	14.8
4.1-5 cm	3	15.7	6	8.4	0	0
5.1-6 cm	2	10.5	3	4.2	0	0
> 6 cm	1	5.2	0	0	0	0
Total	19	100	71	100	27	100

^*^ A: Involved margin; B: Narrow margin; C: Free margin.

**Table 3:** Correlation between clinical and histopathology characteristics and surgical margin status (satisfactory or unsatisfactory).

Margin status*	Unsatisfactory		Satisfactory		*p*-value
	N	%	N	%	
Gender					0.4953
Male	65	72.2	17	62.9	-
Female	25	27.7	10	37.0	-
Differentiation grade					0.4792
WD + MD	69	76.6	22	81.4	-
NVD	14	15.5	2	7.4	-
NVN invasion	18	20.0	4	14.8	746
T-stage					0.0064
T1+T2	61	67.7	26	96.2	-
T3+T4	29	32.2	1	3.7	-
N-stage**					0.6241
N +	30	33.3	7	25.9	-
N-	60	66.6	20	74.0	-
Surgical approach					17
TO	59	65.5	24	88.8	-
Complex	25	27.7	1	3.7	-
µ tumor diameter ± SD***	2.55 ± 1.44	-	1.97 ± 0.99	-	0.0208
µ tumor thickness ± SD***	1.16 ± 0.82	-	0.69 ± 0.46	-	0.0002
Total	90	76.9	27	23.0	-

^*^ A: Involved margin; B: Narrow margin; C: Free margin;

^**^ N+/N-: Histopathology with positive or negative nodes;

^***^ Mean values in cm + standard deviation; NVN: Neurovascular or nodal; TO: Transoral; WD: Well-differentiated; MD: Moderately differentiated; NVD: Not very differentiated.

Patients operated with broad free margins had their tumors resected more commonly through transoral approaches. Despite the lack of statistical significance, patients with unsatisfactory margins were more frequently observed in cases of mildly differentiated tumors, neurovascular invasion, and neck metastasis. Satisfactory resections were more commonly seen in female patients. Women had earlier stage disease and tumors of smaller mean diameter and thickness than males.

## DISCUSSION

Two large multi-center randomized trials confirmed that surgical margins affect the prognosis of patients with SCC of the oral cavity^19^, ^20^, ^21^. These tumors relapse more often^22^ and reduce medium and long term patient survival^4^, ^22^. Liao et al.^8^ analyzed 827 patients submitted to surgery for SCC of the oral cavity, 344 of which with tumors of the tongue and floor of the mouth. In multivariate analysis, tumor thickness and free margins were correlated to local disease control.

Larsen et al.^7^ noted that the rate of free margins decrease as tumor diameter and thickness increase. Only 3% of the tumors with thickness greater than 1 cm had broad free margins. Studies have shown that tumors of the tongue resected with narrow or involved margins resulted in worse local and regional control than tumors involving other sites in the mouth, even when adjuvant therapy was offered^23^. Unlike Byers et al.^24^, who suggested that negative margins have little positive impact for patients with large tumors, Kwok et al.^25^ found that free margins should be sought regardless of tumor size.

Some authors have questioned whether the choice of surgical approach correlates with the rate of narrow and involved margins, given that larger tumors may have been unsuccessfully treated through transoral approaches. Binahmed et al.^13^ did not confirm this assumption, although the study grouped tumors affecting varied oral sites together. Likewise, in our series we found a reverse correlation between transoral resection and unsatisfactory margins, showing the approach was not necessarily associated with the resulting margin status.

Differently from Nason et al.^26^, this study saw higher rates of free margins in female patients of all ages. Apparently, tumor staging has affected this finding.

A high rate of resections with narrow and involved margins was observed in this study. The experience of international groups seems to agree with this finding, as seen in Branwein-Gensler et al.^27^ and Iseli et al.^28^, in which the same criteria for margin categorization was adopted. Evidently, the proximity of oral cavity tumors to a number of structures which if resected add morbidity or mortality, may result in sub-optimal surgical outcomes. Many cases of SCC of the mouth are diagnosed at later stages of the disease. However, there is an apparent discrepancy between referring patients to surgery and the pathology testing data, given the number of cases of unsatisfactory resection even in tumors of smaller diameter, and particularly the shallower neoplasms.

The definition of narrow margin varies substantially in the literature. For SCC of the oral cavity, however, there is consensus in stating that narrow margins are the ones in which there is tumor tissue less than 5 mm away from the border of the surgical specimen^18^, ^29^. In order to produce satisfactory margins from the standpoint of histopathology, one must bear in mind that the borders of formalin-fixed specimens shrink by approximately 40%-50%^18^. Mucosal margins are usually given preference, although most recurrences involve deep resection margins. The importance of tridimensional injury resection was described by Ravasz et al.^4^. Recurrent disease was not recorded in patients with involved mucosal margins. Nonetheless, positive deep margins, particularly when multiple foci of tumor have been observed, were associated with relapsing disease in 38% and 70% of the cases respectively.

The high rates of unsatisfactory margins have shown that visual inspection and palpation at the time of surgery, as well as traditional imaging methods, fall short of determining tumor borders in the oral cavity. Intraoperative frozen section analysis is also limited, as surgical margins cannot be thoroughly assessed and this analysis can only determine the thickness of the tumor-free margin. High-resolution transoral ultrasonography seems to offer better pre and intraoperative assessment of tumor thick-ness^30^ when compared to CT and MRI scans, particularly for tumors with thicknesses under 5 mm^31^. Animal model studies with fluorescent markers hold some promise in accurately identifying tumor borders^32^.

## CONCLUSION

Despite the correlation between larger tumors and unsatisfactory resection margins, as also observed by other authors, the rates of narrow and involved margins are also high in tumors with diameters of two centimeters and less. Higher complexity surgical procedures were not associated with better resection margins. Apparently, traditional methods used to assess tridimensional tumor diameter before and during surgery and the proximity of tumors to vital structures still pose significant obstacles to obtaining proper oncologic margins.
